# Using a support vector machine to determine loyalty in African, European, and North American telecoms

**DOI:** 10.3389/frma.2022.1025303

**Published:** 2022-12-21

**Authors:** Clene Mohlala, Felix Bankole

**Affiliations:** School of Computing, University of South Africa, Pretoria, South Africa

**Keywords:** brand science, computing, brand engineering, loyalty, support vector machine, telecommunications, data science

## Abstract

Brand loyalty is seen as a repeat purchase and the ability to recommend services or products. Telecommunication service providers require loyal customers to stay in business. The current study examines the impact of brand function and corporate image on customer loyalty in the telecommunication industry. The research employed a total of 971 responses from an anonymous online survey of telecommunication customers in Africa, Europe, and North America. Employing partial least squares, the study examined the relationships between brand function, corporate image, and loyalty. The result showed that brand function and corporate image have a significant positive effect on customer satisfaction. In addition, a machine learning algorithm was used to model the best prediction for consumer recommendations of products and services provided by their telecommunication service provider to friends and family.

## Introduction

The telecommunication industry has grown worldwide due to the increasing demand of people wanting to stay connected to loved ones or businesses seeking telecommunications infrastructure to have a competitive advantage over their competitors. Globally, there are about 5.1 billion mobile users (Kemp, 2019). AT&T, Verizon Wireless, and Rogers Communications are three of the top North American telecommunication service providers (Editor, 2019), while MTN, Orange, and Orascom are leading African telecommunication service providers (Minnock, 2019). British Telecom UK (United Kingdom) and Vodafone are two of Europe's top telecommunication service providers (O'Dea, 2019). In Africa, there are ~213 telecommunication service providers, while Europe has about 830 mobile operations, and there exists around 130 mobile operators in North America (Issa and Jha, 2019). With such fierce competition throughout the continents, one may ask, what sets apart the individual operators within each continent? What creates a uniqueness that either attracts or retains customers in such a robust and competitive environment? Is it the brand? What constitutes a brand in telecommunications? Furthermore, why do brands matter?

Brands are economically beneficial to organizations to enable consumers in finding products belonging to such organizations (Zimmer and Kapferer, 2013). For instance, when consumers identify a specific brand, they can already decide on the product. This decision-making approach creates brand awareness that allows consumers to lower their search costs based on preferences or the brand quality/attributes of the product being acquainted with. Hence, customers have reasonable expectations about the product or service they are looking for.

This research aimed to examine the impact of brand function and corporate image on loyalty in telecommunication service providers in Africa, Europe, and North America.

## Research background

In the current circumstances where the competition for customers among telecommunications service providers is fierce, one is bound to ask what sets apart one service provider from another so that they gain new customers and at the same time retain their existing customers rather than lose them to their competitors. Loyalty is what keeps existing customers committed to specific brands. Brand loyalty can be achieved by focusing on what the brands stand for and provides, and it can be achieved through customer satisfaction, attachment, and association. This relational research evaluates the effect of brand function and corporate image in the telecommunication industry. Therefore, the research question deliberated upon in this research is to investigate the impact of brand function and corporate image on customer loyalty in the context of Africa, Europe, and North America. The main objective is to understand the relationship between constructs such as:

- Brand function and service quality.
- Service quality and value, image, satisfaction, culture, loyalty.
- Culture and satisfaction, loyalty, and image.
- Image and value, loyalty, and satisfaction.
- Value, satisfaction, and loyalty.

We hereby provide hypotheses based on the above-stated constructs to examine if there are significant relationships between them.

## Research contribution

This study provides a comparative analysis of how brand function and corporate image create loyalty in three continents, namely, Africa, Europe, and North America. It introduces a two-step approach using a conceptual model derived from structural equation modeling and machine learning algorithms. The conceptual model is formulated through existing research that does not focus on brand function and culture, such as Lam (2007), Hopkins et al. (2009), Hu et al. (2009), Lai et al. (2009), Hapsari et al. (2016) and Gantsho and Sukdeo (2018). The study by Lai et al. (2009) focuses on Chinese telecommunication providers, while Lam (2007), Hopkins et al. (2009), Hu et al. (2009), Lai et al. (2009), Hapsari et al. (2016) and Gantsho and Sukdeo (2018) focus on satisfaction, value, image, culture, service quality, and loyalty. The current study extends the extant literature by adding brand function as a construct and including multiple continents to provide a holistic view on how brand function and corporate image create loyalty among telecommunications service providers. Further, the study adds machine learning to evaluate algorithms that predict loyalty, which is demonstrated either by remaining with the same mobile operator and/or by being able to recommend the products and services of their mobile operator to friends and family.

## Concept and terms

### Branding

Branding differentiates the goods of one producer from those of another competing producer. This differentiation acts as a strategic tool for decision-making. This could be in the form of a corporate culture based on how it portrays that culture externally to its customers (Ropo, 2009). It is also an interrelated complex system (Franzen and Sandra, 2008) that allows one producer to prominently project their products from that of their fellow competitors (Kotler, 1992). Keller and Lehmann (2006) explain that brands are financial vehicles for an organization.

### Brand function

The function of a brand is to illustrate the nature of the product, service, experience type, or brand-provided benefits, while the descriptive modifier serves to distinguish the nature of the product or service (Franzen and Sandra, 2008) from others. Though brands are built based on a product, they serve multiple purposes for an organization with their basic purpose being a marketer for their organization. As for the customer, brands simplify their choice and reduce risk by offering a product of a certain quality and trust (Keller and Lehmann, 2006). Increasing information efficiency, reducing risk, and creating value-addition or image benefit are some of the important brand functions in the business sector (Sugawara and Nikaido, 2014).

### Corporate image

Every organization has its own image, which is perceived to be the mental picture of that organization by its stakeholders (Adeniji et al., 2014). Corporate image and reputation are linked and therefore act as indicators that organizations use to increase brand equity (Heinberg et al., 2018). Corporate image can be referred to as an image of what an organization represents (Roberts-Bowman and Walker, 2020).

### Telecommunications

Telecommunications is the electronic exchange of information in the form of data, voice, and video through technologies like fiber optics, using instruments such as fixed telephones or mobile, radio, television, and internet (Otsetova and Georgiev, 2018). There are two categories of telecommunications—fixed-wire and mobile (Whalley and Curwen, 2012).

- Fixed-wire relates to telecommunications that are offered *via* cable to homes and organizations, for example, fiber optics.
- Mobile telecommunications is the services offered to consumers wirelessly, for example, mobile networks and radio.

### Brand loyalty

Brand loyalty is a commitment to make repeat purchases (Farida and Ardyan, 2018). Loyalty can be explained as the ability to retain customers through different means that resonate with the customers and can be physical or emotional. Furthermore, loyalty can be determined through brand resonance (Franzen and Sandra, 2008; Zimmer and Kapferer, 2013). The most crucial driver of brand equity is the achievement of brand loyalty (Sugawara and Nikaido, 2014).

### Conceptual model

The study by Lai et al. (2009) showed that loyalty could be achieved in a telecommunication organization by testing relationships between quality, value, satisfaction, image, and loyalty. The conceptual model for this study was drawn from the study of Lai et al. but was modified by including brand function and culture for testing relationships in Africa, Europe, and North America. Venetis and Ghauri (2004) and Tsoukatos (2007) studied service quality and customer retention and found that service quality had a significant positive effect on customer retention, and customer retention was an indication of loyalty. Hew et al. (2017) examined the relationship between brand attachment (a loyalty status) to smartphone repurchase and found that brand attachment had a significant positive influence on the repurchase of the same brand of smartphones.

In their study, Hopkins et al. (2009) found that service scripts would be effective if there were no cultural differences between service employees and customers. Meanwhile, Hu et al. (2009) focused on quality, perceived value, corporate image, and customer satisfaction. Their study concluded that:

- service quality, perceived value, and customer satisfaction are statistically significant by having a positive impact;
- service quality, perceived value, customer satisfaction, and corporate image are statistically significant by having a positive effect; and
- service quality, perceived value, corporate image, and behavioral intentions have a positive impact as service quality and behavioral intentions are not significant.

**Figure 1:**
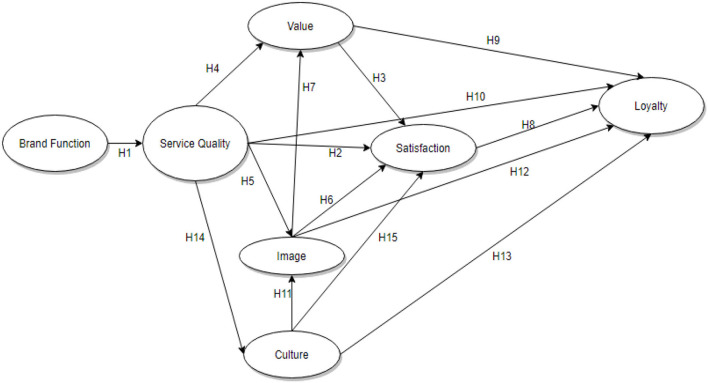
Conceptual model.

The study revealed that service quality had an indirect effect on behavior through satisfaction and corporate image. Lam (2007) found that individuals who score high as individualists are likely to switch brands in his study on the effects of culture on brand loyalty. Similarly, Gantsho and Sukdeo (2018) focused on culture and service quality and concluded that culture had a positive effect on service quality. This literature was used in formulating this study's conceptual model as follows:

Figure 1 illustrates the conceptual model of the relationships which were used to formulate the hypotheses. Khattak et al. (2020) used a questionnaire survey to collect data across 54 continents, this study followed suit and employed a survey across 3 continents.

### Hypotheses development

H1. A brand function has a significant and positive effect on service quality.

A brand function illustrates the nature of the product, service, experience, or brand-provided benefits. At the same time, the descriptive modifier distinguishes the nature of the product or service (Franzen and Sandra, 2008) from others. Brands are built based on a product, at the same time brands serve multiple purposes for an organization with their basic level being a marketer for their organization. As for the customer, brands offer them simplicity in making a choice and the ability to reduce risk by purchasing a product of a certain quality and trust (Keller and Lehmann, 2006). The brand function is measurable on a profile of multiple attributes or characteristics; therefore, it is defined as the perception of the ability to utilize a product or service based on its physical or non-physical performance. It is the determination of needs and/or wants that the brand must satisfy for its customers. It also identifies functions that the brand should fulfill in the lives of its customers and consumers and the values that the brand should uphold.

H2. Service quality has a significant and positive effect on customer satisfaction.

Perceived quality and perceived value are the top two determinants of customer satisfaction (Fornell et al., 1996).

H3. Perceived value has a significant and positive effect on customer satisfaction (Karjaluoto et al., 2012).

Perceived quality and perceived value are the top two determinants of customer satisfaction (Fornell et al., 1996). Perceived value is positively influenced by perceived value whereas price will influence value negatively (Lai et al., 2009).

H4. Service quality has a significant and positive effect on perceived value.

Previous studies (Hapsari et al., 2016; Lin and Huang, 2018) found that the more a customer has a perception of good quality service, the more the customer will perceive value from the corporate. When customers get good quality service, it enhances their perception of the benefits received. Perceived quality is the perception the customer or consumer has about the product or service in comparison to other products, and this is the belief of the customer or consumer about the product or service. Moreover, this belief influences the customer or consumer's attitude toward the brand. Perceived quality and product quality are two separate constructs; the first is based on the customer, while the latter is based on product build quality which depends on the functionality of the product such as ease of use and ease of maintenance (Zimmer and Kapferer, 2013).

H5. Service quality has a significant and positive effect on corporate image.

A customer's evaluation of service quality, value, and satisfaction can be drawn from the corporate image drawn by the corporate. This image creates a void of satisfaction which the service quality will fill once the customer experiences good service quality (Hu et al., 2009; Lai et al., 2009).

Therefore, a relationship exists between previewed value, service quality, corporate image, customer satisfaction, and customer loyalty. The general cohort theory suggests that contextual social and economic backgrounds influence the values, attitudes, and behavior of people of the same generation (Lin and Huang, 2018).

H6. A corporate image has a significant and positive effect on customer satisfaction.

A positive image contributes to a good experience while consuming the service or product and makes it more pleasurable—socially and emotionally (Lai et al., 2009). Zimmer and Kapferer, (2013) state that the favorability of brand association is achieved by persuading the consumers or customers that the product or its attributes are relevant. Furthermore, its benefits are satisfying customer needs and bringing customers peace of mind and joy.

H7. A corporate image has a significant and positive effect on perceived value.

Brand attributes are descriptive features that characterize a product or branded service, while brand benefits have personal value and meaning which the customers are attached to when they consider the product or the brand's service (Zimmer and Kapferer, 2013).

Quality, value, and satisfaction can directly lead to customer loyalty (Hu et al., 2009; Lai et al., 2009), thus:

H8. Customer satisfaction has a significant and positive effect on loyalty.
H9. Perceived value has a significant and positive effect on loyalty (Karjaluoto et al., 2012).
H10. Service quality has a significant and positive effect on loyalty.
H11. Culture has a significant and positive effect on corporate image.

Branding is not only about physical culture but also about corporate culture; for instance, how corporates brand themselves internally through their culture and how they relay that culture externally to set it apart from other companies is considered branding (Ropo, 2009).

H12. A corporate image has a significant and positive effect on customer loyalty.

Brand architecture maximizes the transfer of equity throughout the brand and its individual products and services to improve the process of trial and repeat purchase (Zimmer and Kapferer, 2013).

H13. Culture has a significant and positive effect on customer loyalty.

Culture can have a significant influence on an individual's thinking and behavior, but individuals with high individualism are less prone to switching brands (Lam, 2007).

H14. Service quality has a significant and positive effect on culture.

There exists an important relationship between culture and service quality (Gantsho and Sukdeo, 2018).

H15. Culture has a significant and positive effect on customer satisfaction.

Hopkins et al. (2009) suggest that, when culture is an important matter, it may interfere with service scripts which play a significant role in determining customer satisfaction and the overall service experience.

### Research methodology

The research employed a quantitative method using a structural equation model and a machine learning algorithm. The research audience was aimed at people over 18 years of age in Africa, Europe, and North America who used telecommunication products. The survey was sent out online with no bias or targeting of specific regions, ethnic groups, or countries; it was aimed at each continent. Only the top mobile operators of those continents were available for selection; however, the “Other (not listed)” option was included for any unlisted mobile operator for each continent.

### Data collection

The research used an anonymous survey to collect behavioral data from participants from the three continents. The survey was created on an online platform called Survey Monkey and was distributed *via* their channels. The same link to the survey was circulated on social media platforms.

### Summary

This research made use of realistic measurable properties in the form of a survey questionnaire, with specific questions to examine certain dimensions of the constructs/variables found in the conceptual model. Second, the research was conducted through an anonymous online survey. Lastly, the research seeks to validate a theory that is set out in a group of hypotheses.

SEM was used to examine the relationship between the constructs/variables as per the conceptual model. Once the relationships were validated to have a significate positive effect on each other as per the conceptual model, the survey data were inserted into a supervised ML to evaluate which ML algorithm can make a better prediction on the two loyalty columns. This two-step approach of SEM and ML was used by Al-Skaf et al. (2021) in a study involving 350 students examining the students' acceptance of social media in education along with its factors. Al-Skaf et al. (2021) used SEM to evaluate the theoretical model, while ML was used to reinforce the theoretical model developed and make predictions based on the collected survey data. In another study by Shi et al. (2018), SEM, ML algorithm, and Random Forest was used for evaluating the main contributing factors on vegetation carbon stocks, direct and indirect total effects of the main deriving factors on above-ground carbon stocks, and changes in standardized effects from 2004 to 2014.

## Data results

### Data reliability and validity

Construct validation was conducted by exploratory factor analysis using WarpPLS 7.0, while construct reliability was achieved using Cronbach Alpha coefficiency testing. A Cronbach alpha value of 0.7 and higher represents good reliability (Kock, 2015). Appendix A contains the results of the Cronbach alpha coefficients, which are above 0.7. Table 1 shows the results on the survey participants by continent and combined where else, Tables 2, 3 presents the survey participants by gender across and age groups respectively by continents and combined. Table 4 shows the selected mobile operator by participants.

**Table 1:** Demographic.

**Africa**	**Europe**	**North America**	**Combined**
343	421	207	971

**Table 2:** Gender.

**Gender**	**Africa**	**Europe**	**North America**	**Combined**
Male	149	204	127	480
Female	189	212	76	477
Other	5	5	4	14

**Table 3:** Age group.

**Age Group**	**Africa**	**Europe**	**North America**	**Combined**
18–25	79	114	71	264
26–33	119	134	69	322
34–41	63	83	29	175
42–49	38	51	17	106
50+	44	37	20	101
Other		2	1	3

**Table 4:** Mobile operator.

**Operator**	**Responses**
AT&T mobility	61
BT (UK)	33
Maroc Telecom	1
MegaFone	51
MTN	154
Orange	46
Orange (France)	14
Orascom Telecom	1
Other (Not listed)	121
Rostelecom	24
Safaricom	9
Sprint Corporation	32
Telecom Italia (Italy)	12
Telkom	62
T-Mobile US	78
Verizon Wireless	71
Vodacom	135
Vodafone (UK)	66

## Demographic analysis of respondents

### Analysis

Structural Equation Modeling (SEM) is a multivariate statistical framework employed to model complex relationships between direct and indirect variables (Elston et al., 2012). Partial Least Squares (PLS) is a method of constructing predictive models in which factors are more than one and highly collinear (Urbach and Ahlemann, 2010; Kante et al., 2018).

### SEM constructs

Formative constructs are defined by causality flow from indicators to the construct (Roni et al., 2015).

SEM is chosen because it can evaluate hypothesized relationships among variables by a two-step approach of confirmatory factor analysis, which confirms the significance of the data, and structural model evaluation, which evaluates the validity of the model. Saura and Ribeiro-Soriano (2021) explains that a P < 0.05 demonstrates statistical significance in data.

### SEM regression analysis results

The results of hypothesis testing are significant at a *p-*value of < 0.05 and highly significant at a *p-*value of < 0.01. The model's fitness and quality are determined by the average block VIF (AVIF) value and are acceptable if ≤5 and ideally ≤3.3. The average full collinearity VIF (AFVIF) value is acceptable if ≤5 and ideally ≤3.3.

A total of 971 responses were received. The data showed good reliability with a Cronbach alpha loading of more than 0.7, which is acceptable. The model was tested for fitness and quality with average path coefficient (APC) = 0.305, *P* < 0.001, average r-squared (ARS) =0.490, *P* < 0.001, and average adjusted r-squared (AARS) =0.489, *P* < 0.001 and average block VIF (AVIF) =2.167 and average full collinearity VIF (AFVIF) =2.572.

**Figure 2:**
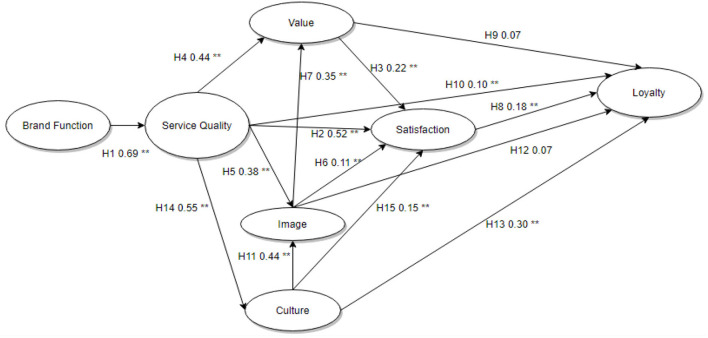
SEM results. *P < 0.05; **P < 0.01; ***P < 0.001.

Figure 2 shows the *P-*values obtained among the relationships after regression analysis. Table 5 illustrates the results of the regression testing of SEM. Validated indicates that the relationship was found to be significant, whereas rejected implies that the relationship was not significant.

**Table 5:** Comparison of SEM vs. multiple regression (MR) (Nusair and Hua, 2010; Alavifar et al., 2012; Westland, 2015; Connolly, 2020).

	**Structural equation modeling**	**Multiple regression**
Features	Multivariate statistical analysis	Linear regression
	Factor Analysis	Evaluates interactions among multiple variables
	Evaluates paths of hypothesized relationships among variables	Employs multiple regression which bridges gaps between correlation and analysis on variances when it comes to addressing the research hypothesis
Advantages	Allows multiple regression equations	More than one predictor variable
	Incorporates latent variables	More precise understanding of the association of each factor
	Accounts for errors in measurements in the estimation process	Accounts for all important factors in the model
Type of variables for evaluation	Independent variable and dependent variables	Independent variable and dependent variables
Approach/technique	Two steps: confirmatory factor analysis and structural model evaluation	Linear regression and non-linear regression

Most of the proposed hypotheses were proven; only two hypotheses (H9 and H12) were rejected.

### Predictive modeling

Machine Learning (ML) is an artificial intelligence tool that allows a computer system to learn from experience, examples, and data by using algorithms (Dhanda et al., 2019). It is used to solve real-life problems and for experimental work (Anifowose et al., 2016; Hangtwenty, 2018). Table 5 provides a comparison of SEM and multiple regression features, advantages and techniques.

### ML usage

The same data used in the structural equation modeling to validate the hypotheses was input into machine learning to establish a model that can make predictions. Prediction models were tested using Rapid miner 9.7.002. Rapid Miner has been used earlier (Ngwenya, 2019) for mining and predicting nicotine usage. The prediction was based on one column [LTY2], which was: “I am more likely to recommend the products and services of my current mobile telecommunication operator to friends and relatives.”

### Supervised machine learning

A supervised method of machine learning was selected to perform the test. The factor scores generated from the SEM analysis were used in the SVM model.

### Prediction modeling

A total of 971 responses are fed into the prediction models in Rapid Miner with the following characteristics:

A total of 67.5% are willing to recommend their current mobile operator's products and services to friends and relatives.

Europeans were more likely to recommend their mobile operators' products and services, followed by Africa and North America.

### Comparison of models

Saura et al. (2022) used clssifiers in their study on Twitter data; Support Vector Classifier, Multinominal Naïve Bayes, Logistic Regression and Random Forest Classifier. The SVM scored an accuracy of 88.13% with a standard deviation of 2.42%. The model took 2 min and 6 s to train.

The [LTY1] for ‘Agree' weighed much more, followed by [LTY1] for ‘Neither Agree Or Disagree'. This shows that the two loyalty questions are tied together. A decision on [LTY2] is impacted by a decision on [LTY1]. Satisfaction, function, value, and image are represented in the top ten weights, meaning that they have an impact on loyalty for [LTY2] with regard to the SVM model.

## Support vector machine—production model

Kernel Model.
Total number of support vectors: 339.
Bias (offset): 0.113.
Feature weight calculation is only possible for two class learning problems.
Number of classes: 3.
Number of support vectors for class Agree: 128.
Number of support vectors for Neither Agree Or Disagree: 140.
Number of support vectors for class Disagree: 71.

**Figure 3:**
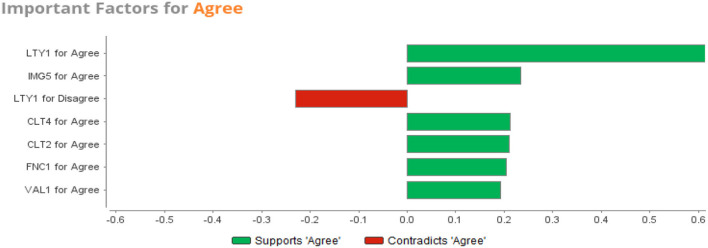
SVM important factors for agree.

Figure 3 illustrates the top weights for SVM for Agree on [LTY2] prediction.

Predictions are used to anticipate the future through controlled modeling from data patterns. Accuracy and speed play important roles in predictive modeling. A desirable model/algorithm is accurate with a reasonable time taken to train the model. The SVM achieved 88% predictive accuracy which shows a standard level of the predictive scenario. SVM uniquely assures a balanced predictive performance (Pisner and Schnyer, 2020). Similarly (Ma et al., 2012) employed deep learning with multiple classifiers to improve accuracy of classification.

## Discussion

The research aimed to examine the impact of brand function and corporate image on loyalty in telecommunication service providers in Africa, Europe, and North America. This study was a continuation of Lai et al. (2009).

The objective of the research was to examine if there were significant positive effects between the below-stated relationships, which could lead to customer loyalty:

- Brand function and service quality.
- Service quality and value, corporate image, satisfaction, culture, and loyalty.
- Culture and satisfaction, loyalty, and corporate image.
- Corporate image and value, loyalty, and satisfaction.
- Value, satisfaction, and loyalty.

The study added two new constructs which were not part of Lai et al.'s (2009) study. The newly added constructs added five new relationships which were hypothesized and evaluated.

The study used SEM which evaluated hypothesized relationships among variables by a two-step approach of confirmatory factor analysis. It confirmed the significance of the data, and structural model evaluation evaluated the validity of the model. Table 6 provides the outcome of the SEM results which proved that there was indeed a significant and positive effect on the eleven sets of hypotheses out of the fourteen hypotheses set out in the conceptual model. While Table 7 illustrates the number and percentage of responses for all continents. Table 8 illustrates response percentages by all continents.

**Table 6:** Hypotheses testing results.

**Hypothesis**	**Remark**
H1. A brand function has a significant, positive effect on service quality.	Validated
H2. Service quality has a significant, positive effect on customer satisfaction.	Validated
H3. Perceived value has a significant, positive effect on customer satisfaction.	Validated
H4. Service quality has a significant, positive effect on perceived value.	Validated
H5. Service quality has a significant, positive effect on corporate image.	Validated
H6. A corporate image has a significant, positive effect on customer satisfaction.	Validated
H7. A corporate image has a significant, positive effect on perceived value.	Validated
H8. Customer satisfaction has a significant, positive effect on loyalty.	Validated
H9. Perceived value has a significant, positive effect on loyalty.	Rejected
H10. Service quality has a significant, positive effect on loyalty.	Validated
H11. Culture has a significant, positive effect on corporate image.	Validated
H12. A corporate image has a significant, positive effect on customer loyalty.	Rejected
H13. Culture has a significant, positive effect on customer loyalty.	Validated
H14 Service quality has a significant, positive effect on culture.	Validated
H15. Culture has a significant, positive effect on customer satisfaction	Validated

**Table 7:** [LTY2] Distribution.

**Combined–[LTY2]**	**No of responses**	**Response percentage**
Agree	655	67.5%
Neither agree or disagree	237	24.4%
Disagree	79	8.1%

**Table 8:** [LTY2] Continent distribution.

**[LTY2]**	**Africa**	**Europe**	**North America**	**Combined**
Agree	61.80%	76.6%	59.4%	67.5%
Neither Agree Or Disagree	25.07%	19.5%	33.3%	24.4%
Disagree	13.11%	4.5%	7.2%	8.1%

After the data was validated and verified through SEM and Cronbach alpha, the data was then run through different machine learning algorithms and SVM came up first as the most accurate model among other models. Table 9 illustrates the accuracy of each model along with its standard deviation run on the combined content data. Table 10 illustrates the top 10 weighted attributes of the best performing model (SVM). Table 11 illustrates the performance of the SVM model, where else Table 12 illustrates SVM's confusion matrix results.

**Table 9:** [LTY2] Model performance comparison.

**Model**	**All Combined—[LTY2]**	
	**Accuracy**	**Standard deviation**
Decision tree	83.05%	3.15%
Deep learning	84.83%	2.11%
Fast large margin	86.69%	1.02%
Generalized linear model	83.46%	1.88%
Gradient boosted trees	84.90%	2.68%
Logistic regression	75.91%	3.67%
Naive bayes	80.88%	2.24%
Random forest	86.68%	2.76%
Support Vector Machine	88.13%	2.42%

**Table 10:** Top 10 SVM weights.

**Attribute**	**Weight**
LTY1 for agree	0.50
LTY1 for neither agree or disagree	0.21
STF5 for agree	0.18
FNC1 for agree	0.14
IMG4 for agree	0.13
VAL1 for agree	0.12
VAL2 for agree	0.11
IMG2 for neither agree or disagree	0.11
CLT13 for agree	0.10
CLT8 for agree	0.10

**Table 11:** SVM model performance.

**Criterion**	**Value**	**Standard deviation**
Accuracy	88.1%	+-2.4%
Classification error	11.9%	+-2.4%
Profits from model	212	
Profits for the best Option (Agree)	102	
Gains	110	

**Table 12:** SVM model confusion matrix.

	**True neither agree or disagree**	**True disagree**	**True agree**	**Class precision**
Predicted neither agree or disagree	59	11	2	81.94%
Predicted disagree	10	178	8	90.82%
Predicted agree	1	1	8	80.00%
Class recall	84.29%	93.68%	44.44%	

This research added to the body of knowledge by adding five new relationships to the original conceptual model of Lai et al. (2009). These are as follows:

1. A brand function has a significant, positive effect on service quality.
2. Culture has a significant, positive effect on corporate image.
3. Culture has a significant, positive effect on customer loyalty.
4. Service quality has a significant, positive effect on culture.
5. Culture has a significant, positive effect on customer loyalty.

All the above sets of hypotheses were found to be true. These results add to the literature on brand science and engineering knowledge.

## Conclusion

This study aimed to examine how brand function and corporate image create loyalty in North America, Europe, and Africa. It sought to determine if there were significant positive effects between (1) brand function and service quality (2) service quality and value, corporate image, satisfaction, culture, and loyalty (3) culture and satisfaction, loyalty, and corporate image (4) corporate image and value, loyalty, and satisfaction, and (5) value, satisfaction, and loyalty. A conceptual model was drawn, and fifteen hypotheses were developed.

A total of 971 responses were received *via* an anonymous online survey. PLS regression was used to test the construct validity for each continent and the data combined.

After the hypotheses were evaluated and validated in their majority, the same data were used for regression analysis to evaluate the best possible prediction model from a range of machine learning algorithms. The predictions were based on the two loyalty questions which were set out in the survey. The hypotheses need to be validated first before the introduction of predictive modeling because if the majority of the hypotheses were not validated, then the predictions would be based on an invalidated concept.

## Research results

The regression analysis proved that (1) brand function has a significant and positive effect on service quality, (2) service quality has a significant and positive effect on customer satisfaction, (3) service quality has a significant and positive effect on perceived value, (4) service quality has a significant and positive effect on corporate image, (5) corporate image has a significant and positive effect on perceived value, (6) culture has a significant and positive effect on corporate image, and (7) service quality has a significant and positive effect on culture in all three continents and combined. This substantiates the importance of service quality, brand function, and corporate image as key driving factors.

## Key contributions

Other studies had not considered the purpose of brand function. This study examined the effect of brand function on service quality since brand function identifies with those brands that pride themselves on service quality to obtain loyalty or preference among customers who are looking for a quality product or service. Other studies acknowledged culture as affecting loyalty. However, these studies did not examine the effect of culture on corporate image or the effect of culture on value. In this study, the effect of culture has been examined concerning customer satisfaction, value, and image, as well as loyalty.

## Limitations

This study was conducted *via* an anonymous survey and was limited to online responses. Online anonymous surveys have the challenge of not being able to ensure that a single person does not take the survey more than once. One cannot be sure that if the same survey was to be sent out to the same people, which are unknown, the same survey results could be obtained. No formal interviews were done during this survey. Due to virtual private networks, there is no 100% certainty that the surveys were done by people living in the continents because virtual private networks can mask a person's location to another continent. Regardless, the Survey Monkey platform targeted users from different continents based on their criteria; therefore, the data were trusted to be coming from the three continents.

## Recommendations

Telecommunications managers must be aware that their brand is a tool that is used to communicate with customers. Therefore, when a customer sees their brand, it needs to resonate with them, and customers must get a service that meets their expectations, if not more. Managers need to consider that loyalty is not defined by just one concept of service delivery or culture; it is a complex feature that must be continuously worked on. Therefore, the recommendation to telecommunications managers is to create a system that can monitor the favorability of their brand by its customers and prospectus customers by maintaining a quality of service that customers can be proud of. This system must include as many dimensions/constructs of branding as possible like brand image, service quality, value, brand attachment, brand awareness, brand judgment, and customer satisfaction. Managers must always look at branding holistically and over time adjust branding dimensions in which their organization may be lagging in to keep their customers happy and attract new ones.

Future research should consider a mixture of online surveys and interviews. More focus can be placed on brand awareness, brand attachment, service performance, and brand equity as they are variables within brand engineering.

## Data availability statement

The original contributions presented in the study are included in the article/Supplementary material, further inquiries can be directed to the corresponding author/s.

## Ethics statement

Ethical review and approval was not required for the study involving human participants in accordance with the local legislation and institutional requirements. Written informed consent to participate in this study was not required from the participants in accordance with the national legislation and the institutional requirements.

## Author contributions

CM conducted the research under guidance, mentorship, and supervision of FB. Both authors contributed to the article and approved the submitted version.
