# Supplementary material for: Using a support vector machine to determine loyalty in African, European, and North American telecoms
Source: Front Res Metr Anal. 2022 Dec 21;7:1025303. doi: 10.3389/frma.2022.1025303 (PMC9811816; doi:10.3389/frma.2022.1025303)
Supplement: Supplementary file 1 [file Data_Sheet_1.doc]

Appendix A: Combined – General SEM Analysis Results


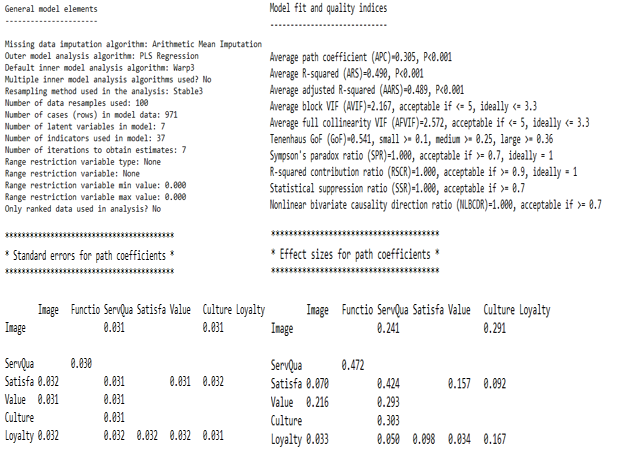


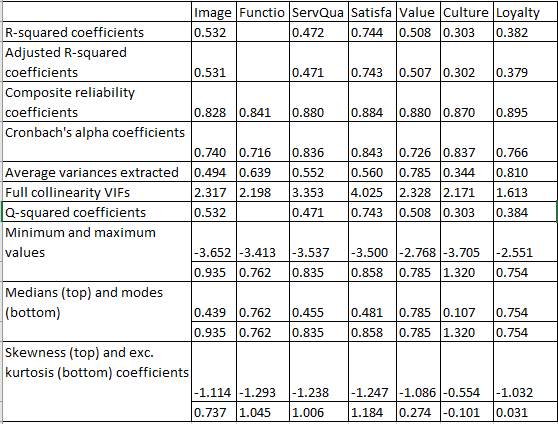


Appendix B: Survey Questionnaire

|  | **1 = Agree; 0.5 = Neither Agree Nor Disagree; 0= Disagree** |  |  |  |
| --- | --- | --- | --- | --- |
| **Image** |  |  |  |  |
| 1 | [IMG1] I find mobile services (voice, data, and coverage, etc.) of my telecommunications service provider unique from other providers. | 1 | 0.5 | 0 |
| 2 | [IMG2] I can clearly remember my mobile telecommunication operator on top of my head. | 1 | 0.5 | 0 |
| 3 | [IMG3] When I think of telecommunication services and products my telecommunications operator comes to mind first. | 1 | 0.5 | 0 |
| 4 | [IMG4] My telecommunication operator has a better reputation than its competitor. | 1 | 0.5 | 0 |
| 5 | [IMG5] My telecommunication operator has good prestige. | 1 | 0.5 | 0 |
| **Satisfaction** |  |  |  |  |
| 1 | [STF1] The mobile services (voice, data, and coverage, etc.) offered by my telecommunications provider gives me confidence and dignity. | 1 | 0.5 | 0 |
| 2 | [STF2] The mobile services (voice, data, and coverage, etc.) of my telecommunication operator make me feel good. | 1 | 0.5 | 0 |
| 3 | [STF3] The mobile services (voice, data, and coverage, etc.) I get from the telecommunication operator that is suitable for me. | 1 | 0.5 | 0 |
| 4 | [STF4] The mobile services (voice, data, and coverage, etc.) of my telecommunications give me a sense of good experience and satisfaction. | 1 | 0.5 | 0 |
| 5 | [STF5] I am satisfied with my telecommunication operator. | 1 | 0.5 | 0 |
| 6 | [STF6] Overall, I am satisfied with my telecommunication operator. | 1 | 0.5 | 0 |
| **Function** |  |  |  |  |
| 1 | [FNC1] There exists a dignified relationship between me and my mobile telecommunications provider in the form of function and purpose on voice, data, and coverage, etc. | 1 | 0.5 | 0 |
| 2 | [FNC2] The mobile services (voice, data, and coverage, etc.) from my telecommunications provider are practical to my needs. | 1 | 0.5 | 0 |
| 3 | [FNC3] The mobile services (voice, data, and coverage, etc.) I get from my telecommunications provider is reliable. | 1 | 0.5 | 0 |
| **Service Quality** |  |  |  |  |
| 1 | [SQL1] I think that the service quality I get from my telecommunications operator for voice, data, and coverage is good. | 1 | 0.5 | 0 |
| 2 | [SQL2] The response I get from my telecommunications provider (reporting problems, fault finding, resolution, etc.) is responsive. | 1 | 0.5 | 0 |
| 3 | [SQL3] I am happy to pay more for the delivery of mobile services (voice, data, coverage) from my telecommunication service provider. | 1 | 0.5 | 0 |
| 4 | [SQL4] I am happy with the overall service quality (security, cost, delivery, etc.) from my telecommunications operator. | 1 | 0.5 | 0 |
| 5 | [SQL5] The service quality I get from my telecommunications service provider meets my expectations. | 1 | 0.5 | 0 |
| 6 | [SQL6] Overall, the service I receive from my telecommunication operator for mobile services (voice, data, cost, security, and coverage, etc.) is valuable. | 1 | 0.5 | 0 |
| **Value** |  |  |  |  |
| 1 | [VAL1] The service quality I receive from my telecommunication operator is worth my time, energy, and efforts. | 1 | 0.5 | 0 |
| 2 | [VAL2] I am proud to be associated with my telecommunication operator. | 1 | 0.5 | 0 |
| **Culture** |  |  |  |  |
| 1 | [CLT1] I often talk about my telecommunication operator benefits (free SMS, data, and free voice minutes) with my peers. | 1 | 0.5 | 0 |
| 2 | [CLT2] Using telecommunication services is a personal decision. | 1 | 0.5 | 0 |
| 3 | [CLT3] I use telecommunication services less often than I need to. | 1 | 0.5 | 0 |
| 4 | [CLT4] I feel comfortable when I try to use mobile telecommunication services (voice, data, and coverage, etc.). I find it clear and simple to use. | 1 | 0.5 | 0 |
| 5 | [CLT5] I am conversant/familiar/up-to-date with the use of mobile telecommunication services. | 1 | 0.5 | 0 |
| 6 | [CLT6] I like to show a firm statement about my character and individuality. | 1 | 0.5 | 0 |
| 7 | [CLT7] Mobile Telecommunication services are highly efficient and will improve my quality of life. | 1 | 0.5 | 0 |
| 8 | [CLT8] I place great value on mobile services (voice, data, and coverage, etc.) and the functions that can be achieved by using telecommunication services. | 1 | 0.5 | 0 |
| 9 | [CLT9] I like being in competition with others. | 1 | 0.5 | 0 |
| 10 | [CLT10] I place great value on material success. | 1 | 0.5 | 0 |
| 11 | [CLT11] I like to depend on others. | 1 | 0.5 | 0 |
| 12 | [CLT12] I normally agree to the expectations or suggestions of others who are seen as important or influential (e.g. my boss/celebrity). | 1 | 0.5 | 0 |
| 13 | [CLT13] There is a strong association between social influences (e.g. family, friends, boss, or employer) and my intention to use telecommunication services. | 1 | 0.5 | 0 |
| **Loyalty** |  |  |  |  |
| 1 | [LTY1] I am more likely to switch to another mobile telecommunications provider in the future. | 1 | 0.5 | 0 |
| 2 | [LTY2] I am more likely to recommend the products and services of my current mobile telecommunication operator to friends and relatives. | 1 | 0.5 | 0 |
